# The epigenetic landscape of brain metastasis

**DOI:** 10.1038/s41388-025-03315-1

**Published:** 2025-02-27

**Authors:** Aoibhín M. Powell, Louise Watson, Lara Luzietti, Stefan Prekovic, Leonie S. Young, Damir Varešlija

**Affiliations:** 1 School of Pharmacy and Biomolecular Sciences, RCSI University of Medicine and Health Sciences, Dublin, Ireland; 2 Department of Surgery, RCSI University of Medicine and Health Sciences, Dublin, Ireland; 3 Center for Molecular Medicine, University Medical Center Utrecht, Utrecht, The Netherlands; 4 Beaumont RCSI Cancer Centre, Beaumont Hospital, Dublin, Ireland

**Keywords:** Metastasis, Epigenetics

## Abstract

Brain metastasis represents a significant challenge in oncology, driven by complex molecular and epigenetic mechanisms that distinguish it from primary tumors. While recent research has focused on identifying genomic mutation drivers with potential clinical utility, these strategies have not pinpointed specific genetic mutations responsible for site-specific metastasis to the brain. It is now clear that successful brain colonization by metastatic cancer cells requires intricate interactions with the brain tumor ecosystem and the acquisition of specialized molecular traits that facilitate their adaptation to this highly selective environment. This is best exemplified by widespread transcriptional adaptation during brain metastasis, resulting in aberrant gene programs that promote extravasation, seeding, and colonization of the brain. Increasing evidence suggests that epigenetic mechanisms play a significant role in shaping these pro-brain metastasis traits. This review explores dysregulated chromatin patterns driven by chromatin remodeling, histone modifications, DNA/RNA methylation, and other epigenetic regulators that underpin brain metastatic seeding, initiation, and outgrowth. We provide novel insights into how these epigenetic modifications arise within both the brain metastatic tumor and the surrounding brain metastatic tumor ecosystem. Finally, we discuss how the inherent plasticity and reversibility of the epigenomic landscape in brain metastases may offer new therapeutic opportunities.

## Introduction

### Clinical overview, epidemiology, and molecular mechanisms of brain metastasis

The majority of cancer-related deaths arise from metastasis rather than the primary tumor. Brain metastasis (BrM), involving the central nervous system (CNS), represents a critical stage of this process. BrM are secondary tumors that originate from primary cancers elsewhere in the body and spread to the brain. This is distinct from primary brain tumors, such as gliomas and glioblastomas, which originate from cells within the brain itself. Despite its severity, BrM mechanisms remain underexplored, limiting therapeutic options [1]. BrM primarily originates from lung (20–56%), breast (5–20%), and melanoma (7–16%) cancers [2–6], with a rising incidence in renal cell carcinoma (RCC) and gastrointestinal (GI) cancers [7]. Molecular subtype of the primary tumor influences BrM likelihood, as seen in *ALK*-rearranged and *KRAS-*mutated non-small cell lung cancer (NSCLC) [8], or human epidermal growth factor receptor (HER) 2-positive and triple-negative breast cancer (TNBC) subtypes, which manifest earlier metastasis compared to estrogen receptor-positive (ER+) tumors [9–12].

BrM is usually associated with advanced stages of primary disease [2, 13, 14], with factors such as ethnicity, age, and location influencing risk [2, 15]. For example, African Americans show higher BrM incidence for lung, melanoma, and breast cancers, though lower rates for RCC [2, 15]. Younger patients (aged 20–39) are more likely to develop breast cancer BrM, while lung cancer peaks in middle age, and melanoma, RCC, and colorectal cancer BrM appear more commonly after age 50 [2, 15, 16]. Though overall survival has improved, it remains low, with median survival for BrM patients ranging from 7 to 34 months depending on cancer type [17]. Symptoms include seizures, headaches, and cognitive impairment, all of which impact quality of life, and emerging treatments face challenges such as the blood-brain barrier (BBB), which hinders drug efficacy [18–20].

Although BrM mechanisms remain poorly understood, metastatic inefficiency is evident: only a small percentage of circulating tumor cells (CTCs) survive and establish metastases. For instance, only 0.02% of injected melanoma cells formed tumors in a study by Luzzi et al. [21]. Successful metastasis likely depends on molecular and epigenetic factors in both tumor cells and the brain microenvironment. BrM exhibits distinct genomic and phenotypic characteristics compared to primary tumors [11, 22], with frequent molecular subtype switching, particularly in breast cancer [11, 23–27]. Studies have revealed genetic heterogeneity between primary tumors and BrM, [25, 28–31], with BrM from lung adenocarcinoma showing higher amplification rates of *MYC*, *YAP1*, and *MMP13*, alongside deletions in *CDKN2A/B*, compared to the primary tumor [32]. Moreover, BrM often harbors targetable mutations not found in extracranial sites, emphasizing the limitations of single-sample tumor analyses [31]. Genomic alterations in breast cancer BrM often affect pathways involving HER, PI3K, cyclin-dependent kinases, and DNA repair [22, 25, 31]. These discrepancies between BrM and primary tumors may explain variable responses to targeted therapies, with extracranial tumors responding while intracranial disease progresses.

Recent research utilizing transcriptome analysis has uncovered dynamic gene programs in BrM, suggesting that these programs are often driven by epigenetic dysregulation rather than mutations in canonical oncogenes or tumor suppressor genes [25]. Metastatic cancer cells must adapt dynamically to thrive in new metastatic niches, each presenting unique challenges. The immune environments and resident cell types in bone, liver, and lung differ from those in the brain, leading to distinct interactions and adaptation strategies that cancer cells may need to employ [33]. The brain, in particular, poses unique obstacles not encountered in other common metastatic sites, such as the need to penetrate the BBB, navigate a distinct immune landscape, and interact with specialized neural cells [33–35]. As a result, cancer cells metastasizing to the brain may require increased adaptability compared to those metastasizing to other organs. Therefore, findings from studies on primary tumors or other metastatic sites may not be directly applicable to BrM due to these fundamental differences. This heightened adaptability is reflected in the significant transcriptomic and epigenetic changes observed in BrM, enabling cancer cells to modify gene expression programs crucial for overcoming the brain’s unique barriers.

## Fundamentals of epigenetic mechanisms

**Fig. 1:**
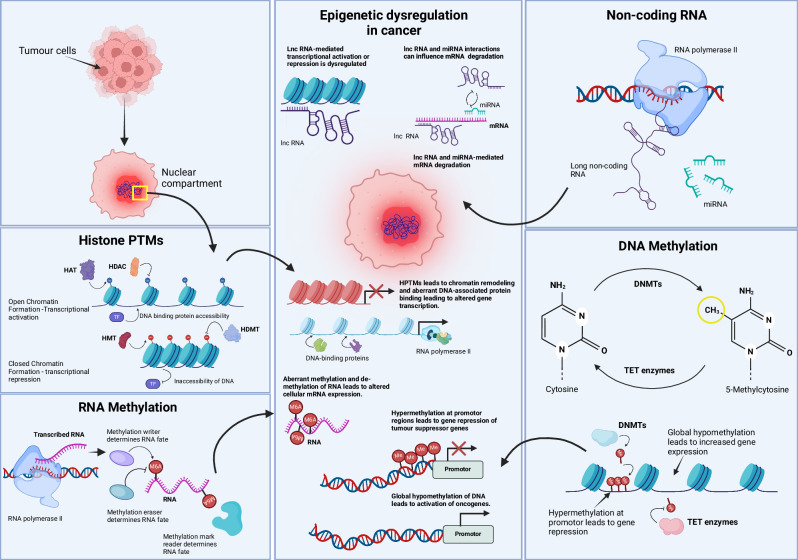
Schematic representing epigenetic mechanisms and their dysregulation in cancer. Normal regulation of epigenetic mechanisms is essential for physiological cell function. However, alterations in histone post-translational modifications (PTMs), RNA methylation, non-coding RNAs, and DNA methylation have been shown to promote oncogenesis. Histone PTMs alter chromatin accessibility and thus control transcription and DNA binding. In cancer, disruption of this process can lead to chromatin remodeling and aberrant gene expression. RNA methylation controls the fate of mRNA, and exploitation of this process can lead to altered mRNA translation. Non-coding RNAs, including miRNAs, are necessary for the post-transcriptional regulation of mRNA. Dysregulation in their expression or function can lead to unwanted oncogenic mRNA translation. Lastly, DNA methylation, a critical epigenetic mechanism for gene expression control through the addition or removal of methyl groups, can be exploited in cancer-specific contexts to silence tumor suppressors or activate oncogenes. In summary, any imbalance in these processes can result in the transformation of a normal cell into a cancer cell, and loss of control over epigenetic mechanisms can further promote metastatic processes. HAT histone acetyltransferase, HDAC histone deacetylase, HMT histone methyltransferase, HDMT histone demethylase, TF transcription factor, m6A N6-methyladenosine, Me methylation, lncRNA long non-coding RNA, miRNA microRNA, DNMT DNA methyltransferase, TET ten-eleven translocation (enzymes).

**Fig. 2:**
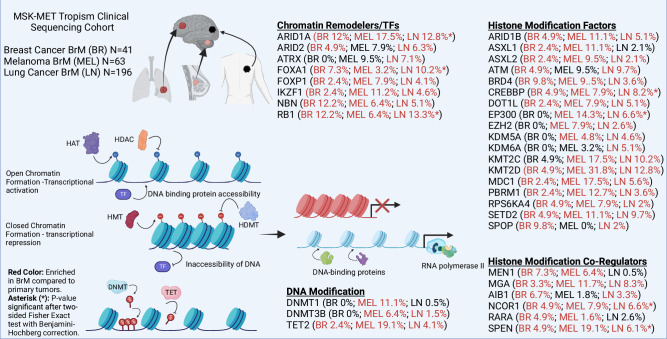
Mutational landscape of epigenetic factors in brain metastatic tumors. This figure highlights the role of key epigenetic regulators that are frequently mutated in BrM. The data presented are derived from the MSK-MET Tropism Clinical Sequencing Cohort [39, 40], which includes 300 BrM from common primary tumor sources, with a focus on breast (BR), lung (LN), and melanoma (MEL). The data were analyzed using cBioPortal to identify frequently altered epigenetic factors across these tumor types. The figure indicates the mutation rates of these epigenetic regulators for each individual source of BrM. Red colored text indicates epigenetic factors enriched in BrM compared to primary tumors. Asterisks (*) denote statistical significance (*p* < 0.05) after a two-sided Fisher’s Exact test with Benjamini-Hochberg correction. The diagram also depicts chromatin in both its open and closed states with various key regulators involved. These regulators modulate chromatin accessibility and gene expression by adding, removing, or reading histone marks and DNA methylation patterns. This figure underscores the critical role of epigenetic dysregulation in the pathogenesis of brain metastasis and highlights the potential for targeting these alterations in therapeutic strategies.

Epigenetic mechanisms modify gene expression without altering DNA sequences [36], and play a pivotal role in regulating cellular functions by controlling DNA accessibility to transcriptional machinery. These heritable changes are crucial for cell differentiation and ensure continuity in cell division. Key epigenetic modifications include DNA/RNA methylation, histone variants and post-translational modifications (HPTMs), and non-coding RNAs (ncRNAs) (Fig. 1), all of which shape gene expression. Dysregulation of these processes can lead to cancer and support the plasticity of metastatic tumor cells [37, 38]. In BrM, dysregulation of epigenetic mechanisms is a key driver of disease progression. Notably, mutations in genes encoding epigenetic regulators themselves can lead to widespread epigenetic changes. Figure 2 highlights the mutational landscape of frequently altered epigenetic regulators in BrM originating from breast, lung, and melanoma. Data from the MSK-MET Tropism Clinical Sequencing Cohort [39, 40] reveals that mutations in epigenetic factors are common across these tumor types and are predominantly enriched in BrM compared to primary tumors. Cancer cells may exploit these changes to adapt to the microenvironment of the metastatic site, modulating gene expression programs to promote survival, proliferation, and immune evasion in the brain.

This review aims to consolidate current knowledge on epigenetic mechanisms in BrM, explore their role in pro-tumorigenic transcriptional programs, and evaluate the potential of epigenetic therapies to combat metastatic growth and improve outcomes.

## DNA methylation

DNA methylation, typically linked with gene silencing, involves adding a methyl group to cytosine at CpG dinucleotides, forming 5-methylcytosine [41, 42] in both gene promoters and repetitive DNA sequences such as transposable elements. This process is mediated by DNA methyltransferases (DNMTs), which act as “writers” by adding methyl groups to DNA. The methylated DNA is recognized by “readers”, such as methyl-CpG-binding domain proteins, which interpret the methylation mark and recruit other factors to modulate chromatin structure and gene expression [43]. Conversely, ten-eleven translocation (TET) enzymes function as “erasers” initiating the removal of methyl groups by converting 5-methylcytosine to 5-hydroxymethylcytosine (5hmC), a key step in DNA demethylation. Disrupted DNA hydroxymethylation patterns have been observed in cancers, suggesting TET proteins act as both cancer promoters and suppressors, highlighting them as therapeutic targets [43]. Typically, unmethylated CpG islands near gene promoters allow transcription factor (TF) binding, while methylation silences genes by restricting TF access. The balance between hypomethylation and hypermethylation is essential for normal cell function, with disruptions leading to cancerous gene activation or silencing [42, 43]. Common cancer-related methylation abnormalities include hypermethylation of tumor suppressors like *TP53* and *BRCA1* and global genomic hypomethylation. Such hypomethylation can lead to the activation of oncogenes, expression of repetitive elements, and a viral mimicry response [44–49]. The efficacy of drugs like temozolomide in cancers with hypermethylated O6-Methylguanine-DNA-methyltransferase (*MGMT*) promoters demonstrates the therapeutic potential of targeting these epigenetic changes [50, 51].

### DNA methylation in brain metastasis

#### Epigenetic changes and methylation patterns in brain metastasis

DNA methylation appears to play a pivotal role in BrM, serving both as a mechanism of tumorigenesis and a potential biomarker for disease prognosis. In a cohort of melanoma BrM specimens, methylation-specific multiplex-ligation probe amplification identified complex patterns of methylation and deletion in genes like *CDKN2A* and *PTEN*, which were independent of *BRAF* and *NRAS* mutations [52]. A significant increase in hypermethylation at high-density CpG areas suggested a role in genomic instability and transcriptional reprogramming during melanoma transition to BrM [53]. Additionally, melanoma BrM-specific partially methylated domains associated with brain function and development were identified, potentially allowing melanoma cells to adapt to the brain microenvironment [53]. These findings support the hypothesis that melanoma cells undergo transcriptional reprogramming to a brain-like phenotype, aiding their survival [53].

Recent studies have also revealed significant methylation changes in NSCLC BrM, such as *ZNF154* promoter methylation, which correlates with poor prognosis and a stem-like phenotype in metastatic cells [54]. Methylome analyses of patient-matched melanoma metastases revealed that intracranial samples shared more similarities with each other than with extracranial ones, indicating patient-specific methylation patterns. This suggests either that the brain microenvironment imposes common epigenetic pressures leading to convergent evolution among metastatic cells or that these similarities, in part, reflect contributions from the surrounding brain tissue. The proximity of tumor cells to various brain-resident cells increases the likelihood of obtaining mixed-cell populations during sample collection. Consequently, methylation and transcriptomic profiles may reflect a combination of tumor-intrinsic changes and signals from the surrounding brain tissue. Despite these limitations, the studies identified key alterations in cytokine and MAPK signaling pathways, which may serve as potential biomarkers for BrM progression [55]. The ability of molecular profiling to distinguish between intra- and extracranial melanoma metastases further underscores the diagnostic value of specific methylation patterns [56].

#### Epigenetic biomarkers for brain metastasis progression

Xu et al. identified differential DNA methylation in NSCLC primary tumors, including *ERBB2* promoter hypomethylation, that later metastasized to the brain [49]. In breast cancer BrM, frequent molecular subtype switching, in addition to *ESR1* downregulation and hypermethylation, highlights the significance of DNA methylation in metastatic progression [26]. Methylation analysis comparing BrM to primary breast tumors and normal tissues revealed genes with altered patterns, detectable in circulating tumor DNA (ctDNA), which could serve as prognostic markers [57]. Additionally, an analysis of unmatched breast to BrM samples (*n* = 32) identified hypermethylation and downregulation of cell migration-related genes (*PENK, EDN3, ITGAM*) and hypomethylation with upregulation of the histone variant *HIST1H2BJ* [58].

Recent studies reported that brain metastatic prostate cancer (PCBrM) exhibits a homogeneous DNA methylation landscape, contrasting with the heterogeneity in primary tumors, suggesting clonal selection of epigenetically altered cells during metastasis [59]. In PCBrM, aberrant methylation was linked to mutational backgrounds and Polycomb repressive complex 2 (PRC2) activity, particularly in *SPOP*-mutant cases, indicating that specific epigenetic reprogramming is necessary for BrM formation [59]. The AURORA US breast study found that 17% of metastases showed DNA hypermethylation or deletions near human leukocyte antigens-A, correlating with reduced immune cell infiltrates in brain and liver metastases, suggesting epigenetic mechanisms in immune evasion [60]. Furthermore, profiling of DNA methylomes from 96 BrM specimens led to the development of the BrainMETH classification system, which distinguishes BrM from primary brain tumors, identifies the tissue of origin, and classifies breast cancer BrM subtypes, offering insights into BrM-specific epigenetic alterations [61–64]. These approaches demonstrate the feasibility of using DNA methylation profiles to accurately identify the primary origins of BrM, which is crucial for treatment selection [63].

#### Epigenetic enzymes and therapeutic targets

DNMT and TET enzymes, key to DNA methylation, can promote tumorigenesis through altered gene expression [43]. Single-cell RNA sequencing (scRNA-seq) of melanoma BrMs demonstrated that non-proliferative cells within the brain express genes associated with the inhibition of *DNMT1* [65]. The suppression of *DNMT1* was influenced specifically through interactions with reactive astrocytes and the brain extracellular matrix. This was shown to not only delay the cell cycle but also activate survival signals like *L1CAM* and *CRYAB*, contributing to the persistence and survival of indolent cancer cells in the brain [65]. Interestingly, DNMT inhibitor RG108 demonstrated a significant antitumor effect by re-expressing hormone nuclear receptor co-activator, *NCOA1*, target genes in hormone receptor-positive endocrine-resistant BrM, underscoring methylation’s reversible role in tumorigenesis [66]. DNMT inhibitors are advancing into clinical trials, highlighting the promise of epigenetic therapies, particularly for cancers like TNBC, where hypomethylation is prevalent [67].

DNA methylation plays diverse roles in cancer development and BrM, offering multiple avenues for therapeutic intervention. The balance of methylation is crucial; both hypo- and hypermethylation can drive cancer progression. Studies highlight the significance of understanding and manipulating epigenetic mechanisms for cancer therapy, emphasizing the need for further research to refine and expand epigenetic-based treatments. Moreover, this also proposes that BrM-promoting epigenetic adaptations could serve as biomarkers for predicting a patient’s risk for CNS metastasis.

## Histone modifications and altered chromatin states in brain metastasis

Chromatin architecture, modulated by DNA-binding TFs, long non-coding RNAs (lncRNAs), chromatin remodeling complexes, and HPTMs, plays a key role in regulating cellular identity, development, and proliferation [68]. Mutations in chromatin regulatory proteins affect 25–30% of cancer driver genes, highlighting the significance of epigenetic alterations in metastasis [69].

DNA histone modifications, along with DNA methylation, control chromatin’s state—either compact heterochromatin or open euchromatin—thus regulating gene expression. DNA is tightly wound around histones (H2A, H2B, H3, H4) in chromatin, and histone variants (e.g., H2A.1, H2A.Z) affect DNA accessibility, influencing cancer progression [70]. Modifications like acetylation and methylation alter chromatin structure. Most chromatin variants found in cancer, identified by characterizing chromatin states from histone post-translational modifications, are distal to genes. Therefore, epigenomic variability critical to oncogenic processes is largely non-coding. Low acetylation and high trimethylation at H4K20 and H3K27 lead to gene silencing, while hyperacetylation and trimethylation at H3K4 and H3K36 are linked to active chromatin [70, 71].

### Enzymes involved in histone modifications and their role in brain metastasis

Histone modifications are regulated by specific enzymes, including writers histone methyltransferases (HMTs), and histone acetyltransferases (HATs), which add methyl and acetyl groups to histone tails, respectively [71, 72]. The erasers in this context are histone deacetylases (HDACs) and histone demethylases (HDMs), which remove these modifications, while bromodomains and chromodomains act as readers that interpret these epigenetic marks [71]. Dysregulation in these enzymes, such as those controlling acetylation and methylation, is widely documented in various cancers, influencing recurrence and survival outcomes [73]. In BrM, the role of these modifications remains underexplored, and it may be critical given several HDAC inhibitors are known to be brain penetrant [74, 75].

Beyond their involvement in microglial development [76], HDACs, such as HDAC2, play roles in chromatin reorganization and tumor stem cell maintenance, interacting with the TGF-β pathway to sustain tumorigenic potential, suggesting HDAC2 as a therapeutic target for primary brain tumors like glioblastoma [77]. These findings imply that similar mechanisms might be relevant in BrM, and thus, HDAC2 could be a prospective therapeutic target worth exploring in the context of BrM. RE1-silencing transcription factor (*REST*) typically binds at RE1/neuron-restrictive silencer elements on target genes to recruit co-repressors such as CoREST and mSin3A, along with HDACs 1 and 2 [78]. Interestingly, in BrM, the functionality of the REST protein is reported to be diminished. This complex represses neuronal gene expression in non-neuronal cells by inducing chromatin remodeling and transcriptional silencing, which is critical for neuronal development, maturation, and maintenance in healthy brain tissue. Reduced *REST* function in the context of BrM results in increased production of synaptic signaling mediators and neurotransmitters, giving breast cancer cells a major colonization advantage [78].

*HDAC8* may modulate chromatin dynamics and gene expression in melanoma BrM by altering H3K27ac levels and enhancing accessibility at *JUN* binding sites through the deacetylation of *EP300*, effectively inactivating it. This shift facilitates increased *EP300* interaction at *JUN*-transcriptional sites while reducing its association with melanocyte-inducing transcription factor sites, promoting melanoma cell invasion and resistance to stress, ultimately leading to enhanced metastatic potential [79]. scRNA-seq data further associated *HDAC8* expression with a neural crest stem cell signature in melanoma BrM, underscoring its role in metastasis [79].

Among the key epigenetic regulators are also histone-lysine N-methyltransferases, *KMT2C* and *KMT2D*. These enzymes are responsible for adding methyl groups to histone H3K4, specifically contributing to the H3K4me1 mark, which is associated with enhancer regions in the genome. Recent study reported that deletion of *KMT2C* or *KMT2D* in non-metastatic breast cancer TNBC models drives BrM through altered H3K4me1, H3K27ac, and H3K27me3 marks, with increased KDM6A binding and *MMP3* upregulation [80]. Targeting the KDM6A*–MMP3* axis via KDM6A inhibition can prevent metastasis in *KMT2C/D* mutant tumors [80]. Some studies also note that circulating histones could be used as a functional biomarker for disease which will be important to study in the context of BrM [73].

## Chromatin and chromatin complexes

While DNA tightly bound to nucleosomes may be less accessible to certain transcription factors and regulatory proteins, it remains accessible to epigenetic machinery. Epigenetic factors such as histone-modifying enzymes (e.g., HATs, HMTs) and chromatin remodelers (e.g., Switch/Sucrose non-fermentable (SWI/SNF) complexes) can interact with nucleosomal DNA and histones [81]. By changing the accessibility of DNA to transcriptional machinery, these interactions result in changes to chromatin structure, such as nucleosome repositioning or histone modification, which in turn controls gene expression. Chromatin remodeling complexes are recruited by epigenetic marks on histones or on the DNA sequence itself e.g., HPTMs, DNA binding proteins, or DNA methylation marks [81]. Given the ability of chromatin remodelers to change the accessibility of DNA and to move histones, the gain or loss of function of a chromatin remodeling complex could have a wide array of downstream effects leading to aberrant gene expression. Additionally, the 3D organization of the genome plays a crucial role in gene regulation and cancer progression. Perturbations in 3D genome organization can promote acquired drug resistance, as changes in genome architecture can alter the accessibility and expression of genes involved in therapy response [82]. For example, *STAG2*, a component of the cohesin complex, is often mutated in cancer and may contribute to metastatic spread and invasion. *STAG2* loss has been shown to rewire oncogenic and developmental programs, promoting metastasis in Ewing sarcoma [83].

### Chromatin remodeling complexes and gene regulation in brain metastasis

Chromatin remodeling complexes, such as SWI/SNF, play crucial roles in regulating gene expression. Mutations in subunits of the SWI/SNF complex promote pro-tumorigenic functions, enhancing the activity of oncogenic TFs like *MYC* and *E2F*, which drive cancer progression [84]. These mutations also deregulate critical cellular pathways, including glycolysis, Hippo, Notch, and Akt signaling, some of which have been implicated in the development of BrM [84]. One of the most frequently mutated genes in the SWI/SNF complex is *ARID1A*, which is also commonly altered in BrM (Fig. 2). *ARID1A* mutations have been linked to endocrine resistance in cancers, particularly in the context of hormone-driven cancers [85]. Additionally, a study on CTCs from breast cancer patients with BrM revealed that 67% of all brain metastases lack ARID1A protein expression [86]. ARID1A loss is most frequently observed in TNBC and ER+ BrM patients. However, this study found no correlation between ARID1A expression and other clinicopathological factors, leaving its role in BrM unclear [86].

In addition to chromatin remodeling complexes, histone-modifying complexes like PRC2 are essential for epigenetic regulation. PRC2 is a histone methyltransferase complex that represses gene expression by catalyzing the trimethylation of H3K27, maintaining a closed chromatin state [87]. PRC2 comprises four main subunits: EED, SUZ12, RBAP46/47, and EZH2, with EZH2 acting as the catalytic subunit which has been implicated in metastasis, particularly in breast, prostate, and lung cancers [87–89]. Targeted inhibitors such as tazemetostat are showing clinical promise by modulating these epigenetic interactions [90, 91]. In BrM, EZH2 phosphorylation induces granulocyte colony-stimulating factor production, promoting neutrophil infiltration and increased Src phosphorylation at metastatic sites [92].

### Transcription factors and chromatin interactions in brain metastasis

Disruptions in TF regulation drive cancer cell plasticity and metastasis by activating aberrant pathways. TFs bind to specific DNA sequences to regulate mRNA transcription and protein synthesis, playing essential roles in cell proliferation and cycle control. The activity of certain TFs can be modulated by DNA methylation (e.g., E2F, AP2) and/or chromatin remodeling, which can influence their ability to bind to target DNA sequences [93]. Epigenetic dysregulation can lead to aberrant activation or repression of TFs, which can be associated with progression to BrM. Indeed, integrated epigenomic analyses have revealed that active chromatin changes at JUN target sites correlate with BrM metastatic potential, demonstrating how epigenetic modifications can regulate TF activity and contribute to BrM [92, 94]. A specific chromatin accessibility signature, associated with poor prognosis, highlighted TFs like Forkhead box, POU domain, and JUN-related factors as drivers of organ-specific metastasis. High JUN expression correlates with BrM relapse in patients, suggesting its importance in BrM and potential as a therapeutic target [94].

*EN1* was identified as a TNBC-specific TF associated with neural features and BrM in breast cancer, leading to poor clinical outcomes [95]. TFs like *MYC* and *SOX2*, vital for cellular reprogramming, also drive oncogenic transformation, underscoring their role in cancer’s aggressive traits [96–98]. Following the known impact of *MYC* amplifications on metastasis and poor survival outcomes [98], further studies have detailed how *MYC* enhances BrM. Specifically, *MYC* stimulates invasive growth, macrophage infiltration, and promotes gap junction formation between metastatic cells and astrocytes by upregulating connexin 43 (*GJA1/Cx43*) [99]. Functional assessments using patient-derived xenograft mouse models have confirmed that overexpression of *MYC*, along with *YAP1* and *MMP13*, significantly increases the incidence of BrM [32].

Recent studies have also highlighted the role of *TGLI1* in activating *SOX2* within BrM, promoting cancer stem cell (CSC) traits, and modifying the tumor microenvironment through astrocyte activation enhancing tumor growth and metastatic establishment [100]. SOX2’s activation by TGLI1 underscores its dual role in initiating cancerous traits and adapting to new metastatic niches, enabling CSCs to interact with and reshape their microenvironment to support tumor progression [100]. Jeevan et al. corroborated this finding in showing that breast cancer cells had increased proliferation and migration when exposed to astrocytic media [101]. This again brings to light the ability of cancer cells to adapt and alter their behavior in order to grow in a new metastatic niche. Further research has highlighted the key roles of TFs, *SOX2,* and *SOX9*, in defining the identity of latency-competent cancer (LCC) cells, with differential expressions impacting the epigenetic landscape and transcriptional activity across lung and breast cancer BrM models [102]. By regulating the WNT inhibitor *DKK1*, *SOX2* promotes a quiescent, immune-evasive state in LCC cells, illustrating how TFs interact with epigenetic changes (H3K27ac and Pol II binding) and cell signaling to influence cancer cell stemness, survival, and initiation of metastasis [102].

### Multi-omic epigenetic profiling in brain metastasis research

RNA-seq and ATAC-seq, have demonstrated their utility in studying BrM by revealing distinct gene expression and chromatin accessibility profiles in CTCs and organ-specific metastases. In BrM, upregulated genes linked with peroxisome activity, oxidative phosphorylation, and neurodegenerative pathways reflect the unique influence of the brain microenvironment on tumor cell behavior [103]. Advanced profiling techniques, such as fixed-tissue ChIP-seq for H3K27 acetylation profiling (FiTAc-seq), could be essential for ongoing research on patient-derived BrM samples as they can provide detailed maps of genome-wide enhancer landscapes, deepening our understanding of epigenetic modifications in the clinical setting [104]. Table 1 summarizes key studies on BrM-related epigenetic changes, covering the primary platforms, methodologies, and mechanisms studied, including DNA methylation and histone modifications.

**Table 1:** Key resources and studies profiling epigenetic alterations in BrM.

Epigenetic focus	Platforms/ Software Used	Sample type and number	Cancer type	Accession number	Paper citation
DNA methylation	DNA methylation profiling using ELISA-seq and sequencing of target libraries using NovaSeq 6000, Illumina	29 patients with CNS metastatic NSCLC and 31 patients with advanced NSCLC	NSCLC with brain metastasis	N/A (data in Supplementary Materials of the paper)	Xu et al. [49]
DNA methylation	Human Methylation 450 K beadchip arrays, Illumina	Parrafin embedded archival tissue (melanoma brain metastasis (*n* = 15) and normal brain tissue (*n* = 91)) Melanoma metastasis patient samples (*n* = 142; primaries - liver (*n* = 11), lung (*n* = 32), brain (*n* = 99)) Array carried out on 14 MBM from the frontal lobe	Brain metastatic melanoma	GSE43414 (and Supplementary Materials of the paper)	Marzese et al. [52]
DNA methylation	Infinium Human Methylation450K, Illumina	Melanoma related patient specimens (*n* = 40)	Brain metastatic melanoma	DNA methylation: GSE44661	Marzese et al. [53]
DNA methylation	Methylation profiling using MeDIP-seq and MRE-seq	FFPE from normal lung, primary lung tumor, and brain metastasis (*n* = 11) and fresh frozen tissue from normal lung, primary lung tumor, and brain metastasis (*n* = 1)	Brain metastatic lung cancer	GSE203216	Karlow et al. [54]
DNA methylation	Infinium Human MethylationEPIC array, Illumina	37 metastases samples from 14 melanoma patients	Brain metastatic melanoma	GSE203152	Kraft et al. [55]
Demethylation of LINE-1	Quantitative bisulfite pyrosequencing using PSQ 96 pyrosequencing, Qiagen	Cell lines; A549, HCT116, MDA-MB468, T-47D, ZR-75-1, HS578T, MCF-7, MDA-MB231, SKBR3, MDA-MB453	Breast cancer	N/A (data in Supplementary Materials of the paper)	Miglio et al. (2018)
DNA methylation	450 K methylation array, Illumina	Fresh frozen patient breast cancer brain metastasis tumor samples (*n* = 30)	Brain metastatic breast cancer	N/A (data in Supplementary Materials of the paper)	Pangeni et al. [57]
DNA methylation	NextSeq2000, Illumina Infinium MethylationEPIC, Illumina	32 melanoma brain mets	Brain metastatic melanoma	Data reported in the paper	Radke et al. [56]
DNA methylation	Infinium Human Methylation EPIC 27 K, Illumina	Fresh frozen patient samples from breast cancer brain metastasis samples (*n* = 23 & *n* = 12) and non-neoplastic samples (*n* = 2, *n* = 8 & *n* = 10)	Brain metastatic breast cancer	Gene expression data: GSE52604: DNA methylation: 10.6084/m9.figshare.855629	Salhia et al. [58]
DNA methylation	Infinium MethylationEPIC 850 K, Illumina	155 tissue samples; primary tumors (*n* = 57), metastases (*n* = 95), normal prostate (*n* = 2), normal brain (*n* = 1)	Brain metastatic prostate cancer	EGAD00010002372	Gallon et al. [59]
DNA methylation	HumanMethylation EPIC array, Illumina NovaSeq6000	Breast cancer tumor samples (*n* = 55)	Metastatic breast cancer	RNAseq: GSE209998 DNA methylation: GSE212375	Garcia-Recio et al. [60]
DNA methylation	Infinium HumanMethylation 450 K, Illumina	FFPE tumor samples from patients (*n* = 96) with a range of primary to brain metastasis; BCBM (*n* = 30), LCBM (*n* = 22), MBM (*n* = 44)	Brain metastasis	GSE108576 GSE44661	Salomon et al. [64]
DNA methylation	Infinium HumanMethylation 450 K, Illumina	Patient tumor samples with a range of primary to brain metastasis; BCBM (*n* = 30), LCBM (*n* = 18), MBM (*n* = 44), primary tumor patient samples; breast and lung (*n* = 4)	Primary and metastatic brain tumors	N/A (data in Supplementary Materials of the paper)	Orozco et al. [61]
DNA methylation	Infinium HumanMethylation 27 K, Infinium 450 K and Infinium 850 K, Illumina	DNA methylation profiles collected	Glioblastoma and brain metastasis	N/A (data in paper)	Liu et al. [63]
DNA methylation	PyroMark Q24, Qiagen	Brain metastatic melanoma cell line (WM266.4)	Brain metastasis	GSE150556 GSE150557 GSE150560	Hirata et al. [65]
SeqCap Epi MeDIPseq ChIPseq	HiSeq2000 v4.0 Illumina	Cell lines (MCF7, LY2, T347)	Advanced breast cancer	RNAseq, MeDIP-seq, SeqCap Epi: GSE99649 ChIP-seq: GSE28987	Ward et al. [66]
DNA methylation	EZ DNA Methylation-Direct Kit, Zymo	Cells isolated from mice with lesions or lung adenocarcinoma tumors	Lung cancer	GSE185615	Xu, et al. (2022)
ChIP-seq H3K27a	Not specified	LLC cells from lung and breast carcinoma	Breast and Lung cancer	GSE72956	Maladi et al. (2016)
ChIP-seq H3K27ac	Novaseq6000 2×150 flow cell	WM164 and 1205Lu cells	Melanoma	BioProject: PRJNA903203	Emmons et al. [79]
ChIP-seq ATAC-seq	Illumina HiSeq2000	MDA-MD-231, MDA-MD-231-BrM2 and MDA-MD-231-LM2 cells	Breast cancer metastasis	GSE129647	Cai et al. [94]
FiTAc-seq (protocol)	Illumina NGS	FFPE clinical samples	Breast cancer brain metastasis	GSE140808	Alba et al. (2020)

This table provides a comprehensive overview of key studies focused on the epigenetic mechanisms underlying BrM, categorized by the primary cancer source. It includes detailed information on the specific epigenetic focus of each study, the platforms and software used for analysis, the sample types and sizes, and relevant accession numbers for data access. This resource is intended to serve as a valuable reference for researchers exploring the epigenetic landscape of BrM.

*CNS* central nervous system, *NSCLC* non-small cell lung cancer, *FFPE* formalin-fixed paraffin-embedded, *WES* whole exome sequencing, *MeDIP-seq* methylated DNA immunoprecipitation sequencing, *MRE-seq* methylation-sensitive restriction enzyme sequencing, *CNV* copy number variation, *HMM* hidden Markov model, *ChIP-seq* chromatin immunoprecipitation sequencing.

## RNA methylation

While DNA methylation has long been recognized as a key player in cancer metastasis, the emerging role of RNA methylation in the progression of advanced disease is gaining significant attention [105]. RNA methylation such as N6-methyladenosine (m6A) is increasingly recognized for its impact on tumor cell adaptation, therapy resistance, and disease progression. At a basic functional level, m6A modifications have been implicated in crucial processes like gene splicing and translational control [106]. m6A RNA methylation involves the addition of methyl groups by writers (e.g., methyltransferansferase complex enzymes METTL3 and METTL14), with these modifications subsequently identified by readers, like YTH domain-containing proteins, that prompt RNA turnover or modification, thereby reducing protein synthesis [107–113]. Conversely, eraser enzymes, including the demethylases FTO and ALKBH5, remove m6A modifications, effectively reversing RNA methylation [114, 115].

An evolving body of work has highlighted the causal role of m6A RNA methylation in sustaining tumorigenesis and promoting stem-like characteristics [116–120]. In the context of breast cancer, the role of m6A regulatory elements, particularly erasers such as *FTO* and *ALKBH5*, in influencing disease advancement remains a subject of debate. These enzymes have been associated with negative outcomes and an increased tendency for metastasis [121, 122]. However, research has also shown that inhibiting *FTO* activity may actually promote the proliferation and invasion of breast cancer cells, an effect that aligns with the induction of epithelial-mesenchymal transition (EMT) [123]. The dynamics of m6A methylation, particularly in transitioning to BrM in ER+ breast cancer, show global methyl gains and specific pathway alterations, implicating stem cell differentiation as a pivotal factor influenced by the altered epi-transcriptome [124]. Moreover, *FTO’s* expression correlates with patient outcomes, and its inhibition is linked with reduced tumor growth in breast cancer BrM models [124]. *YTHDF3*, an m6A reader, is significantly associated with breast cancer BrM, playing a vital role in the BrM cascade and impacting patient survival [125]. Overexpression of *YTHDF3* was found to promote the translation of m6A-enriched transcripts of BrM-related genes, including *ST6GALNAC5, EGFR*, and *CX43/GJA1* [125]. Knockdown studies of *YTHDF3* in mouse models resulted in decreased BrM, highlighting its influence on the translation of key metastatic genes. Increased *IGF2BP3* expression, another m6A reader, has been identified in clinical and experimental BrM of breast cancer, suggesting its potential as a predictor of distant metastasis [126]. *IGF2BP3*’s role underscores the importance of RNA methylation in cancer progression and offers a new avenue for therapeutic strategies targeting the epi-transcriptome to mitigate metastatic disease.

The exploration of m6A RNA methylation in BrM represents a frontier with untapped potential. Despite its critical role in regulating gene expression and influencing cancer progression, the specific impact of m6A modification mechanisms on BrM is only beginning to be unraveled. Several research groups are actively investigating this area, aiming to harness the full utility of m6A epitranscriptomic modifications to deepen our understanding of BrM processes.

## Non-coding RNAs

ncRNAs, including microRNAs (miRNAs) and lncRNAs, are critical epigenetic regulators in cancer biology and their dysregulation plays an important role in cancer [127]. Although they do not code for proteins, they influence gene expression through mechanisms like RNA interference and chromatin modification [127]. lncRNAs are involved in the regulation of gene expression at various levels, including chromatin modification, transcriptional regulation, and post-transcriptional processing. miRNAs, typically 20–25 nucleotides long, regulate gene expression post-transcriptionally by binding to target mRNAs, leading to degradation or translational inhibition.

### Dysregulation of miRNAs in brain metastasis

miRNAs have been extensively studied in the context of BrM and a number of specific miRNAs have been implicated in BrM progression. *PTEN* is suppressed in BrM compared to other metastatic sites, a suppression that is reversible upon migration away from the brain [128]. This regulation is mediated by miRNAs within astrocyte-derived exosomes, and blocking these exosomes can restore *PTEN* levels and inhibit BrM [128]. Additionally, *PTEN* loss increases secretion of CCL2, attracting myeloid cells that enhance the survival and proliferation of brain metastatic tumor cells [128]. Cancer-derived extracellular vesicles (EVs), which mediate cell-cell communication by delivering proteins and miRNAs, could play a pivotal role in pre-metastatic niche formation by initiating the breakdown of the BBB, facilitating tumor entry into the brain [129]. Mechanisms involving miR-181c highlight how brain metastatic cancer cell-derived EVs facilitate BrM by promoting BBB disruption. These EVs downregulate *PDPK1*, leading to changes in cofilin activity and alterations in actin dynamics, ultimately compromising BBB integrity [130]. The organ specificity of these exosome interactions is mediated by the types of integrins expressed on their surface: integrin α6β4 predominantly targets lung cells, while integrins αvβ5 and αvβ3 guide exosomes to the liver and brain, respectively, further emphasizing the role of tumor-derived EVs in promoting organ-specific metastasis and invasion by creating pre-metastatic niches that facilitate tumor progression [131].

miRNAs such as miR-132-3p, miR-199a-5p, miR-150-5p, and miR-155-5p are differentially expressed in breast cancers that progress to BrM, with links to invasion and metastasis pathways, showing promise as prognostic markers [132]. Similarly, in lung cancer BrM, miR-145-5p is consistently downregulated due to promoter methylation, leading to increased levels of *EGFR, OCT-4, and MYC*, which enhance tumor migration. Restoring miR-145-5p or depleting its targets significantly reduced metastasis in lung cancer models [133].

### lncRNAs in brain metastasis

Some metastasis-promoting lncRNAs are upregulated in BrM. In NSCLC, *HOTAIR* expression is elevated in BrM compared to primary tumors, although its direct role in promoting BrM is not demonstrated [134]. *MALAT1* levels were also shown to be higher in lung cancer patients with BrM, and functional studies indicate that *MALAT1* promotes migration and metastasis of brain-metastatic lung cancer cells by inducing EMT [135]. *BMOR* was identified as a key brain-enriched lncRNA that facilitates breast-to-BrM by enabling evasion of immune-mediated killing within the brain microenvironment. Mechanistically, BMOR was found to bind to and inactivate interferon regulatory factor 3 in breast cancer cells, and silencing *BMOR* effectively suppressed the development of BrM in vivo [136]. Similarly, elevated expression of lncRNA, Lnc-BM, has been identified as a prognostic marker for BrM progression in breast cancer patients. Lnc-BM was found to promote BrM by activating the JAK2/STAT3 pathway, enhancing vascular co-option and macrophage recruitment in the brain microenvironment [137]. These studies illustrate how lncRNA mechanisms are linked to BrM but as the current investigations are limited, further research is necessary to fully elucidate the functions of lncRNAs in the context of BrM and realize their potential.

## Epigenetic control over the tumor microenvironment

The tumor ecosystem comprises the cellular and tissue surroundings in which a tumor develops. Tumor cells dynamically adapt to and exploit the brain’s tumor ecosystem to foster a conducive niche for metastasis, interacting extensively with the brain’s resident cells. Distinct patterns of immune cell infiltration characterize the BrM tumor ecosystem, varying between primary tumors and metastases, and among different metastatic origins. For example, melanoma BrM predominantly attracts T lymphocytes, whereas breast cancer BrM shows greater neutrophil infiltration, reflecting their role in the inflammatory response [138]. Microglia, neurons, and endothelial cells are important in the context of BrM but the epigenetic regulation of these cells in BrM is currently less well-described in the literature compared to astrocytes which we discuss in this review. For comprehensive reviews covering these components in detail, readers are referred to recent works [33–35, 139].

Astrocytes, particularly in their reactive state, play a dual role in BrM by supporting and impeding cancer progression [140–142]. They become hypertrophic and highly secretory, releasing molecules like plasminogen activators. Cancer cells counteract these by expressing anti-plasminogen activator serpins, such as neuroserpin and serpin B2, which are critical for the onset of BrM [141]. Moreover, reactive astrocytes may be forced into an immunosuppressive phenotype upon interaction with cancer cells. Heiland et al. showed that transcriptional reprogramming of reactive glial cells led them to produce cytokines of anti-inflammatory nature including IL-10 and TGF-B and this was dependent on JAK/STAT pathway activation [140]. Additionally, reactive astrocytes secrete various cytokines such as *CCL7*, *IL-23*, Brain-Derived Neurotrophic Factor (*BDNF), HGF, IFNA*, and *TNFA*, which can either promote or inhibit metastasis, influenced by epigenetic modifications [141, 142].

Astrocytes significantly influence the brain microenvironment. For instance, astrocyte-derived exosomes containing miR-19a can induce *PTEN* loss in cancer cells, leading to increased CCL2 secretion and the recruitment of myeloid cells, thereby promoting metastasis [128]. These interactions are further regulated epigenetically; the expression of inflammatory cytokines such as *CCL7* and *IL-6* is controlled by specific patterns of histone and DNA methylation at their promoters [143]. Similarly, *BDNF*, essential for CNS development and implicated in BrM, is regulated through histone modifications at its promoter, linking epigenetic changes directly to tumor biology [144, 145]. Moreover, astrocytes modify signaling pathways critical for cancer cell survival and metastasis progression. Activation of *PPARG* by molecules secreted by astrocytes supports cancer cell viability, with *PPARG* regulation tightly controlled by epigenetic mechanisms [146, 147]. Phosphorylated STAT3 in reactive astrocytes fosters a pro-tumor environment by enhancing the secretion of oncogenic factors like EGF, TGF-α, and MIF, while elevated PD-L1 expression in reactive astrocytes may suppress cytotoxic T cell activity, thereby facilitating tumor evasion of immune surveillance [148].

Microglia, alongside astrocytes, mediate immune surveillance in the brain microenvironment. Cancer cells exploit microglial phenotypes; notably, an increased ratio of M2 (immunosuppressive) microglia correlates with BrM [149]. Loss of lncRNA, XIST, in cancer cells promotes BrM in breast cancer models by inducing EMT and activating c-MET. These XIST-low cells secrete microRNA-503, transforming microglia toward the M2 phenotype [150]. BrM cells expressing lncRNA, RP11-355I22.7, show reduced response to astrocyte-secreted pro-apoptotic Fas ligand. They secrete CCL2 to recruit myeloid cells, creating a feedback loop where macrophage-secreted IL-6 and oncostatin M activate the JAK/STAT/RP11-355I22.7 axis, promoting tumor growth. This exemplifies how altered expression of a single lncRNA can affect both cancer cells and the tumor ecosystem [137]. The interplay between various cells in brain and cancer cells underscores the significant impact of the microenvironment on BrM, mediated by epigenetic regulations. Epigenetics’ role in shaping the brain metastatic ecosystem highlights promising avenues for developing novel treatments [140].

## Conclusions and future directions

In the context of BrM, it is essential to recognize the importance of epigenetic regulatory mechanisms as they play a vital role in sustaining transcriptional programs specific to BrM that enable tumor cells to adapt to the brain environment. Notably, despite originating from diverse primary tumors, BrMs often exhibit convergent phenotypic and epigenetic adaptations. This suggests that the BrM tumor ecosystem may impose common selective pressures that shape the evolution of metastatic cells. The BrM tumor ecosystem demands significant adaptation from invading cancer cells, leading to shared epigenetic and transcriptomic alterations, thus crucially shaping the evolution of metastatic cells beyond their cell of origin.

Comprehensive multi-omics approaches—including genomic, transcriptomic, and epigenomic analyses—are essential for uncovering molecular drivers and potential therapeutic targets. Single-cell methods, such as scRNA-seq and single-nucleus CUT&Tag could significantly enhance our ability to study epigenomic evolution at the individual BrM cell level. For instance, Gonzalez et al. analyzed over 100,000 cells from human BrMs, identifying functional cell programs and delineating BrM archetypes shaped by tumor-immune interactions [151]. This approach provides a foundational framework for understanding BrM, and studies like these will benefit from integrating epigenomic aspects to better understand how these programs are regulated in uncovering tumor cell-intrinsic and host environmental traits. Moreover, utilizing single-cell DNA methylation profiling with techniques like single-cell reduced representation bisulfite sequencing, combined with spatial epigenomic technologies, will provide a comprehensive view of the epigenetic landscape within BrM. By adopting these advanced methodologies more broadly, we can gain critical insights into BrM progression and develop more effective, targeted therapies.

### Therapeutic implications and future directions

The plasticity and reversibility of the epigenomic landscape in BrM offer unique therapeutic opportunities as dynamic epigenetic modifications can be pharmacologically targeted. HDAC inhibitors, including vorinostat and quisinostat, are capable of crossing the BBB and have shown antitumor activity in vivo [74, 75]. Additionally, targeting EZH2 controlled signaling can reactivate silenced genes and impede BrM progression [92], offering another promising therapeutic approach but current approved therapies lack BBB penetrance. Targeting DNMT1, in particular, may disrupt survival mechanisms in BrM cells, aiding in the elimination of dormant tumor cells [65]. RG108 has shown antitumor effects by re-expressing target genes in endocrine-resistant BrM organotypic models [66] but does not enter the brain at sufficient concentrations due to the BBB. Given the breadth of studies on DNA methylation highlighted in this review, this area appears underutilized and warrants further exploration. Targeting RNA methylation is an emerging novel therapeutic strategy. FTO inhibitors have been shown to reduce tumor growth in breast cancer BrM models and FTO inhibitor meclofenamate is currently being evaluated as a treatment for BrM (NCT02429570).

It is evident that for effective epigenetic therapy, BBB penetration is essential. Strategies such as nanoparticle-based drug delivery, BBB-translocating peptides, and combining epigenetic drugs with agents that transiently disrupt BBB integrity may further enhance drug concentrations in BrM. Finally, combining epigenetic therapies with treatments targeting both genetic and epigenetic alterations as well as immunotherapies, or other targeted agents could enhance anti-tumor responses and provide a comprehensive treatment strategy for BrM.

**Fig. 3:**
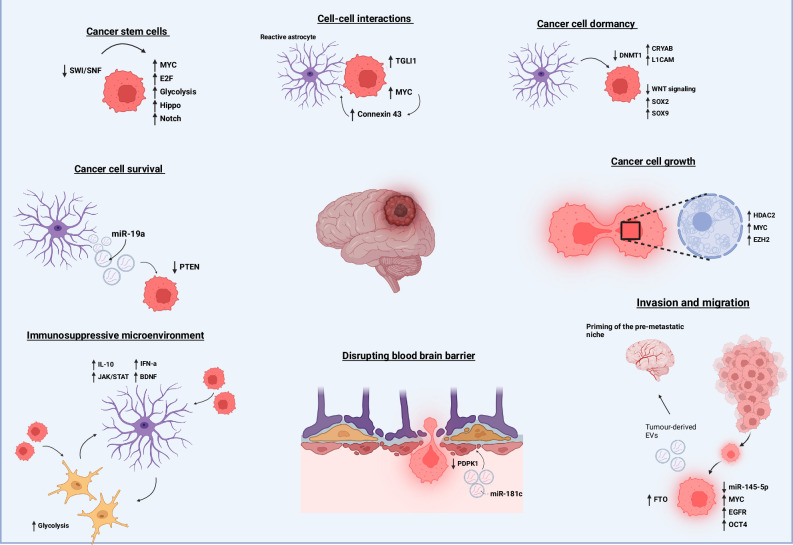
Schematic representing critical epigenetic determinants of key brain metastatic processes. Brain metastasis is the result of a highly complex series of events, all of which are subject to epigenetic control. Any imbalance in these epigenetic mechanisms can promote brain metastatic processes. CSCs, which initiate metastatic homing, can be driven by the upregulation of genes in the Notch and Hippo pathways through the SWI/SNF complex. Primary tumor-derived EVs, influenced by RNA demethylase *FTO*, can prime the brain metastatic niche. Contents of these EVs, such as miR-181c, can facilitate degradation of the BBB by downregulating *PDPK1* in endothelial cells, allowing metastatic cells to pass into the brain. Once in the brain, metastatic cells interact with their microenvironmental niche. Resident astrocytes can promote cancer cell dormancy by triggering *DNMT1* downregulation, which increases the expression of *L1CAM* and *CRYAB* while downregulating Wnt signaling. Metastatic cells and reactive glial cells induce transcriptional reprogramming of astrocytes, promoting a more immunosuppressive, pro-tumorigenic microenvironment through the production of *IL-10, IFN-α*, and *BDNF*. These pro-tumorigenic astrocytes can further support cancer cell growth through direct junctions with cancer cells, such as connexin 43, leading to the upregulation of *MYC* and *TGLI1*, or through astrocyte-secreted molecules like miR-19a, which triggers *PTEN* loss in cancer cells. Supported by all aspects of the brain microenvironment, metastatic cancer cells can proliferate uncontrollably to form metastases, a process further promoted by epigenetic modulators, *EZH2* and *HDAC2*. Abbreviations: SWI/SNF switch/sucrose non-fermentable, EVs extracellular vesicles, FTO fat mass and obesity-associated protein (RNA demethylase), miR microRNA, PDPK1 3-phosphoinositide-dependent protein kinase-1, DNMT1 DNA methyltransferase 1, L1CAM L1 cell adhesion molecule, CRYAB crystallin alpha B, IL-10 interleukin-10, IFN-α interferon-alpha, BDNF brain-derived neurotrophic factor, EZH2 enhancer of zeste homolog 2, HDAC2 histone deacetylase 2, PTEN phosphatase and tensin homolog.

In conclusion, the intricate interplay of epigenetic mechanisms plays a pivotal role in the progression of BrM, influencing every stage from initial metastatic homing to the eventual outgrowth of metastases. These processes, as illustrated in Fig. 3, highlight how key epigenetic determinants contribute to the complexity and resilience of brain metastatic disease. Advancing our understanding through cutting-edge technologies and multidisciplinary approaches will lead to significant advancements in the diagnosis, treatment, and management of BrM. Targeting the interplay between epigenetic modifications and the brain tumor ecosystem holds promise for developing effective therapies to overcome this formidable challenge in cancer treatment.
